# Associations of Body Composition and Physical Function With Incident Diabetes in Older Adults: A 14‐Year Prospective Cohort Study

**DOI:** 10.1002/jcsm.70297

**Published:** 2026-04-20

**Authors:** Ting Zhang, Shuyi Li, Yafei Wu, Jason Leung, Alice Pik‐Shan Kong, Amany K. Elshorbagy, T. W. Auyeung, Timothy Kwok

**Affiliations:** ^1^ Department of Geriatrics Renji Hospital, Shanghai Jiao Tong University School of Medicine Shanghai China; ^2^ Department of Medicine and Therapeutics, Faculty of Medicine The Chinese University of Hong Kong Hong Kong China; ^3^ Jockey Club Centre for Osteoporosis Care and Control The Chinese University of Hong Kong Hong Kong China; ^4^ Hong Kong Institute of Diabetes and Obesity, Prince of Wales Hospital The Chinese University of Hong Kong Shatin Hong Kong China; ^5^ Department of Pharmacology University of Oxford Oxford UK; ^6^ Department of Physiology, Faculty of Medicine University of Alexandria Alexandria Egypt; ^7^ Jockey Club Institute of Ageing The Chinese University of Hong Kong Hong Kong China

**Keywords:** aging, body composition, branched‐chain amino acids, diabetes mellitus, physical performance

## Abstract

**Background:**

Longitudinal associations between age‐related changes in body composition, physical performance and incident diabetes mellitus (DM) in older adults and the mediating role of branched‐chain amino acids (BCAAs) remain understudied.

**Methods:**

This prospective cohort study included 4000 community‐dwelling older adults at baseline. Longitudinal analysis of incident DM involved 3421 participants without baseline DM. Incident DM was identified through self‐reported diagnosis, medication use, hospital records or fasting glucose ≥ 7.0 mmol/L. Body composition (PBF [percent body fat], FMI [fat mass index], ASM [appendicular skeletal muscle mass]) was measured using DXA. Associations of baseline body composition with incident DM were assessed using Cox regression in the full cohort, with sensitivity analysis accounting for competing risk of death. Associations in long‐term subgroups and analysis of 14‐year and prediabetes changes were evaluated using multivariable logistic regression. Serum BCAAs were quantified by LC–MS for mediation analysis.

**Results:**

Among 3421 participants (median age 72 years [IQR 68–76]; 50.1% female) without baseline DM, higher baseline adiposity (PBF: HR 1.613; FMI: HR 1.684; waist: 1.587) and ASM/ht^2^ (HR 1.561) were consistently associated with increased DM risk in adjusted Cox models (all *p* < 0.001). Conversely, higher relative muscle mass (ASM/weight: HR 0.603; ASM/BMI: HR 0.631; both *p* < 0.001) was associated with a lower risk. Faster gait speed was also linked to lower DM risk (HR 0.748, *p* < 0.001). Sensitivity analysis accounting for competing mortality demonstrated consistent results. In the complete 14‐year follow‐up subgroup (*n* = 937, 130 incident DM cases), key baseline associations were confirmed using logistic regression (e.g., ASM/weight: OR 0.633, *p* = 0.005). Analysis of 14‐year changes showed that attenuated loss of relative muscle mass (ΔASM/weight: OR 0.657; *p* = 0.005) was correlated with lower DM risk, whereas increased adiposity was correlated with higher risk (∆PBF: OR 1.509, *p* = 0.019). Pre‐DM changes over 0–4 years also revealed that early increases in waist (OR 1.428, *p* = 0.005) and declines in ASM/weight (OR 0.745, *p* = 0.013) were associated with subsequent DM. Serum BCAAs mediated 18.6%–38.2% of associations between baseline body composition and incident DM (all *p* < 0.01). No significant correlation was found between dietary protein intake and serum BCAAs after adjustment.

**Conclusions:**

In older adults, higher baseline adiposity and lower relative muscle mass were associated with long‐term DM risk, while preserved muscle mass and faster gait speed were linked to a lower risk. These associations, partially mediated by BCAAs, advance the understanding of DM risk pathways and highlight the potential clinical relevance of weight‐ or BMI‐adjusted relative muscle mass assessment in this population.

## 1 Introduction

The global population is experiencing a rapid aging trend, with the number of individuals aged ≥ 65 years expected to reach 1.5 billion by 2050 [1]. This demographic shift has brought increasing attention to health challenges in the elderly, particularly diabetes mellitus (DM), a prevalent metabolic disorder with substantial disease burden globally. Recent data show that the worldwide prevalence of DM among adults has more than quadrupled over the past three decades, escalating from approximately 200 million cases in 1990 to over 800 million in 2022 [2], predominantly Type 2 DM (T2DM). Consequently, there is an urgent need for effective early diagnosis and prevention strategies. In this context, identifying modifiable risk factors, especially those linked to age‐related physiological changes, has become a central focus of preventative research. Emerging evidence indicates an association between DM and sarcopenia in older adults, contributing to functional limitations and adverse health outcomes [3, 4]. This underscores the potential importance of body composition, namely, the balance between muscle and fat, as a key area for mitigating DM risk in aging populations.

Building on this, skeletal muscle, as the body's largest insulin‐sensitive tissue, is pivotal for glucose homeostasis [5]. In the context of aging, age‐related muscle loss and increased fat infiltration have been contributors to insulin resistance (IR) [6]. This body composition shift initiates a cascade of metabolic disturbances. Muscle loss reduces resting metabolic rate and total energy expenditure, promoting overall adiposity and visceral fat accumulation [7]. Aging and obesity promote pathological muscular fat accumulation, exacerbating chronic inflammation and ultimately muscle loss [8]. Notably, such adipose tissue expansion is a potent promoter of chronic low‐grade inflammation, a key driver of IR [9]. Thus, the coexistence of muscle loss and fat accumulation may synergistically exacerbate IR, representing a key pathway linking body composition to DM [10]. Importantly, this pathway may be further amplified through dysregulated branched‐chain amino acid (BCAA) metabolism, creating a potential vicious cycle. At the tissue level, pathological intramuscular fat deposition, a risk factor for impaired glucose metabolism and reduced muscle [11, 12], correlates with elevated BCAA levels [13]. Mechanistically, BCAA accumulation impairs glucose oxidation metabolism [14] and exacerbates IR by activation of the mTOR pathway [15], while IR concurrently disrupts BCAA catabolism, creating a vicious cycle that perpetuates elevated BCAA levels [14, 16]. Therefore, BCAA metabolism may not only be linked to body composition but also act as a key metabolic mediator in the progression from sarcopenic obesity to DM.

However, longitudinal evidence linking long‐term changes in body composition and physical performance to incident DM, particularly in older adults aged ≥ 70 years, remains limited. Prior studies have largely involved younger cohorts or had methodological limitations regarding body composition assessment [17, 18, 19, 20, 21]. Moreover, the potential mediating role of metabolic pathways, such as those involving BCAAs, in this association is not well understood. Therefore, this prospective cohort study aimed to investigate these longitudinal associations in an understudied elderly population: (1) associations of baseline body composition and physical performance measures with incident DM and (2) associations of both 14‐year changes and early pre‐DM changes in these measures with incident DM. Furthermore, we also aimed to explore the potential mediating role of serum BCAAs in the relationship between baseline body composition and incident DM.

## 2 Methods

### 2.1 Study Participants

We analysed data from the Mr. OS and Ms. OS (Hong Kong) study, a prospective community‐based cohort investigating osteoporosis, sarcopenia and other health outcomes [22]. At baseline (2001–2003), 4000 Chinese older adults (2000 men and 2000 women) aged ≥ 65 years were recruited through age‐stratified sampling (65–69, 70–74, ≥75 years) and underwent comprehensive follow‐up at 4, 7 and 14 years. For cross‐sectional demographic comparison, participants were stratified by pre‐existing DM status at baseline (Baseline_DM vs. Baseline_Non_DM, Table 1). For prospective analysis of incident DM, we included participants who were in the Baseline_Non_DM group and had body composition data for Cox regression analysis. For the long‐term follow‐up subgroup analysis, we focused on participants who completed the entire 14‐year follow‐up. The study was approved by the joint Chinese University of Hong Kong and New Territories East Cluster Clinical Research Ethics Committees in Hong Kong (reference number [2003.102]). Written informed consent was obtained from each participant.

**TABLE 1:** Baseline characteristics of subjects.^a^

	Baseline_Non_DM (*N* = 3421)	Baseline_DM (*N* = 579)	*p*
Age, year	72 (68–76)	72 (69–77)	0.025
Men, %	49.9	50.6	0.753
Education, %			0.223
No education	21.0	24.0	
Primary or below	50.6	47.7	
Secondary or above	28.4	28.3	
Living alone, %	10.6	12.1	0.290
Smoking status, %			0.103
Never	63.2	64.1	
Past smoking	29.6	31.1	
Current smoking	7.2	4.8	
Drinking status in the past 12 months, %			0.076
None	86.4	90.5	
< 7 drinks/week	11.3	8.1	
7–13.9 drinks/week	1.5	0.7	
14–27.9 drinks/week	0.7	0.5	
≥ 28 drinks/week	0.2	0.2	
Charlson comorbidity index	4 (3–5)	5 (5–6)	< 0.001
Height, cm	156.8 (150.7–163.3)	157.0 (150.8–163.6)	0.593
Weight, kg	57.7 (51.4–64.6)	59.9 (53.6–67.1)	< 0.001
BMI, kg/m^2^	23.4 (21.4–25.6)	24.2 (22.2–26.3)	< 0.001
Waist circumference, cm	86.4 (80.0–92.4)	88.9 (83.8–94.6)	< 0.001
Waist/height	0.55 (0.51–0.59)	0.57 (0.53–0.61)	< 0.001
PASE score	85.4 (60.8–111.3)	84.7 (60.2–110.4)	0.354
Energy intake, kcal/day	1772.0 (1421.3–2222.5)	1604.6 (1335.6–2035.7)	< 0.001
Carbohydrate intake, g/day	248.1 (200.7–309.5)	223.7 (179.7–269.1)	< 0.001
Fat intake, g/day	53.5 (41.3–70.7)	50.8 (40.3–68.5)	0.075
Total protein intake, g/day	70.3 (53.0–94.0)	67.6 (50.8–89.0)	0.055
Statin use, %	5.20	10.36	< 0.001
Corticosteroid use, %	1.28	0.86	0.393
Creatinine, μmol/L	70.3 (58.2–84.3)	70.7 (59.1–84.5)	0.657
Valine, μmol/L	285.6 (256.5–318.9)	310.7 (282.9–340.9)	< 0.001
Leucine, μmol/L	144.9 (129.5–161.7)	159.8 (143.3–177.8)	< 0.001
Isoleucine, μmol/L	77.7 (68.6–87.5)	86.1 (76.3–98.7)	< 0.001
PBF, %	29.47 ± 7.35	29.50 ± 6.36	0.914
FMI, kg/m^2^	6.85 (5.34–8.60)	7.05 (5.70–8.52)	0.037
ASM/ht^2^, kg/m^2^	6.56 (5.88–7.24)	6.74 (6.04–7.49)	< 0.001
ASM/weight, %	28.23 (25.30–30.99)	27.99 (25.39–30.48)	0.184
ASM/BMI	0.703 ± 0.15	0.697 ± 0.13	0.403
Lower limb—ASM/ht^2^, kg/m^2^	4.89 (4.40–5.38)	5.01 (4.50–5.52)	< 0.001
Grip strength, kg	28 (22–34)	26 (22–34)	0.102
Gait speed, m/s	1.01 (0.87–1.16)	0.98 (0.84–1.14)	0.005
Five‐time chair stand, s	12.22 (10.19–14.64)	12.62 (10.45–15.25)	0.006

^a^ Median (interquartile range) or mean ± standard deviation for continuous variables and percentage (%) for categorical variables were shown.

Abbreviations: ASM, appendicular skeletal muscle mass; ASM/ht^2^, ASM/height^2^; ASM/weight, ASM/body weight × 100; BMI, body mass index; FMI, fat mass index; ht, height; PASE, Physical Activity Scale for the Elderly; PBF, percentage of body fat.

### 2.2 Body Composition

Body composition was assessed using dual‐energy X‐ray absorptiometry (DXA). Fat mass, lean mass and bone mass in the whole body, trunk and limbs were quantified by a Hologic QDR 4500‐W device (Waltham, MA, USA). Muscle mass was derived by lean mass minus bone mass. Appendicular skeletal muscle mass (ASM) was calculated as the combined lean muscle mass of four limbs. Waist circumference (WC) was measured at the midpoint between the lowest rib and the iliac crest. In data analysis, ASM was adjusted for body size to generate different indices: height‐adjusted ASM (ASM/ht^2^) and measures of relative muscle mass including weight‐adjusted ASM (ASM/weight) and BMI‐adjusted ASM (ASM/BMI). Key body composition indices were calculated as follows:

$$\begin{array}{c} \text{Percent body}\;\mathrm{fat}\;\left(\mathrm{PBF},\%\right)=\left[\text{body}\;\mathrm{fat}\;\text{mass}\;\left(\mathrm{kg}\right)/\text{body weight}\;\left(\mathrm{kg}\right)\right]\times 100 \end{array}$$

$$\mathrm{Fat}\;\text{mass index}\;\left(\mathrm{FMI},\mathrm{kg}/m^{2}\right)=\text{body}\;\mathrm{fat}\;\text{mass}\;\left(\mathrm{kg}\right)/{\text{height}}^{2}\;\left(m^{2}\right)$$

$$\text{Height}-\text{adjusted}\;\mathrm{ASM}\;\left(\mathrm{ASM}/{\mathrm{ht}}^{2},\mathrm{kg}/m^{2}\right)=\mathrm{ASM}\;\left(\mathrm{kg}\right)/{\text{height}}^{2}\;\left(m^{2}\right)$$

$$\begin{array}{c} \begin{array}{c} \begin{array}{c} \begin{array}{c} \text{Weight}-\text{adjusted}\;\mathrm{ASM}\;\left(\mathrm{ASM}/\text{weight},\%\right)\\ =\left[\mathrm{ASM}\;\left(\mathrm{kg}\right)/\text{body weight}\;\left(\mathrm{kg}\right)\right]\times 100 \end{array} \end{array} \end{array} \end{array}$$

$$\mathrm{BMI}-\text{adjusted}\;\mathrm{ASM}\;\left(\mathrm{ASM}/\mathrm{BMI},m^{2}\right)=\mathrm{ASM}\;\left(\mathrm{kg}\right)/\mathrm{BMI}\;\left(\mathrm{kg}/m^{2}\right)$$

$$\begin{array}{c} \text{Waist}-\text{to}-\text{height ratio}\;\left(\text{Waist}/\mathrm{ht}\right)\\ =\text{waist circumference}\;\left(\mathrm{cm}\right)/\text{height}\;\left(\mathrm{cm}\right) \end{array}$$

### 2.3 Physical Performance and Muscle Strength

Physical performance was assessed through gait speed and five‐time chair stand, all performed with standardized instructions. Gait speed was measured as the time to complete a 6‐m walk at the usual pace. The shorter duration from two trials was used. For the five‐time chair stand, participants completed two trials of rising from a seated position five times consecutively, with the longer time being retained. Handgrip strength was assessed using a calibrated JAMAR dynamometer (Hand Dynamometer 5030JO, Sammons Preston, Bolingbrook, IL, USA). Participants performed two trials with each hand, with the highest value being recorded.

### 2.4 Outcome Ascertainment

Participants with DM at baseline were excluded. Incident DM cases were identified through a composite definition utilizing multiple sources of information in this community‐based elderly cohort: (1) self‐reported physician diagnosis of DM during any follow‐up interview; (2) current use of glucose‐lowering medication(s); (3) a documented diagnosis of DM (using standard ICD codes) in the electronic medical records of the Hong Kong Hospital Authority (HA) system, linked to the study participants; or (4) a fasting blood glucose level ≥ 7.0 mmol/L. The HA diagnosis was based on standard clinical practice guidelines prevailing during the follow‐up period, which typically included measurement of fasting glucose, HbA1c or oral glucose tolerance test as per physician assessment. Participants were considered to have incident DM upon the first occurrence of any of these criteria. For the primary survival analysis (Cox model), participants were followed from the baseline visit until the date of new‐onset DM, date of death or the last date of follow‐up contact, whichever came first. For the long‐term follow‐up subgroup analysis (logistic regression), incident DM was specifically ascertained at the 14‐year follow‐up visit.

### 2.5 Quantification of Serum BCAAs

Fasting serum BCAAs (valine, leucine, isoleucine) were quantified at baseline using liquid chromatography‐mass spectrometry (LC–MS) [23]. Sample preparation included addition of isotopically labelled internal standards, reduction of disulfides with dithioerythritol and protein precipitation using 5‐sulfosalicylic acid. Chromatographic separation was achieved using the Shimadzu LC‐20ADXR Prominence LC system (Kyoto, Japan), Phenomenex Kinetex Core Shell C18 (100 × 4.6 mm, 2.6 μm) and mobile phase of 0.5% formic acid/0.3% heptafluorobutyric acid aqueous solution with acetonitrile gradient. Mass spectrometric detection was performed using positive ion mode multiple reaction monitoring. Quantification was performed using isotopically labelled internal standards with linear calibration curves, and compound identity was verified against reference standards and spectral libraries. For the 14‐year follow‐up samples, fasting serum BCAAs were quantified using ^1^H nuclear magnetic resonance (NMR) spectroscopy on a Bruker AVANCE III 600 MHz spectrometer in a proprietary pipeline by Nightingale Health Limited.

### 2.6 Covariate Assessments

Trained research assistants conducted face‐to‐face interviews using validated questionnaires to collect demographic data (age, sex and education), lifestyle factors and medical history. Smoking status was categorized as never, past or current. Drinking status was based on alcohol consumption in the past 12 months and categorized as none, < 7 drinks/week, 7–13.9 drinks/week, 14–27.9 drinks/week or ≥ 28 drinks/week. Medical history included physician‐diagnosed chronic diseases and current medication use. Comorbidity burden was quantified using the Charlson Comorbidity Index (CCI). For the primary analysis cohort (participants free of DM at baseline), the CCI was calculated without assigning points for DM. Physical activity was assessed using the Physical Activity Scale for the Elderly (PASE), adapted for the Chinese population in Hong Kong.

Anthropometric measurements were measured to calculate body mass index (BMI). Serum creatinine was quantified using LC–MS/MS. Dietary intake was assessed using a validated food frequency questionnaire (FFQ). Nutrient intakes (energy, carbohydrate, fat, protein) were calculated by multiplying food intake by the nutrient content of the specified food portion size. Energy intake adjustment was performed using the residual method. Animal‐derived protein sources included red meat, fish and shellfish, poultry, processed meat, milk products, eggs, animal giblets and others. Plant‐derived protein included vegetables, cereals, fruits, soybeans and nuts/seeds, and others.

### 2.7 Statistical Analysis

Continuous variables were presented as either median with interquartile range (IQR) for non‐normally distributed data or mean ± standard deviation (SD) for normally distributed data. Categorical variables were expressed as percentages (%). Comparisons between the baseline_DM and baseline_Non_DM groups were performed using the Mann–Whitney *U* test, independent samples *t*‐test and chi‐square test (*χ* ^2^ test).

Each body composition and physical performance index was analysed as a standardized z‐score (per SD increase). In the full cohort, associations with incident DM were assessed using Cox proportional hazards regression, reported as hazard ratios (HRs) with 95% confidence intervals (CIs). **A fully adjusted Cox model was used, which included age, sex, PASE score, energy intake, education, living alone, smoking status, drinking status in the past 12 months, Charlson comorbidity index and use of statin and corticosteroid as covariates.** To account for competing risk of death during follow‐up, sensitivity analysis was conducted using Fine–Gray subdistribution hazard models. Sex‐stratified analyses were performed. For the long‐term follow‐up subgroup, we restricted the analysis to the participants who completed the entire 14‐year follow‐up. Multivariable logistic regression was used to evaluate the associations between baseline body composition and incident DM in this subgroup, reported as odds ratios (ORs) and 95% CIs. Similarly, we examined the relationships between 14‐year changes in body composition and incident DM using identical modelling approaches. To assess the potential for follow‐up bias, we compared baseline characteristics of participants who completed the 14‐year follow‐up with those who did not. Furthermore, to examine changes preceding DM onset, we assessed whether changes during the first 4 years (baseline to year 4) in body composition and physical performance predicted incident DM from years 4–14 among participants who were diabetes‐free and had complete body composition data at both timepoints.

The mediating roles of BCAAs in the association between body composition and incident DM was examined using mediation analysis. The mediation model was adjusted for age, sex, PASE score and energy intake. We reported standardized regression coefficients, average causal mediation effect (ACME) and average direct effect (ADE) from adjusted models and estimated the proportion of mediation effect using nonparametric bootstrapping with 10 000 iterations. To further explore the temporal association of BCAAs with DM, we conducted supplementary analyses. First, we compared BCAA levels between individuals with and without DM at baseline and at 14‐year follow‐up (Table S6). Second, we evaluated whether baseline BCAA levels predicted having high BCAA levels at the 14‐year follow‐up (Table S7). **Spearman correlation analysis was performed to assess the associations among dietary protein/meat intake, serum BCAAs and incident DM, adjusting for age, sex, PASE score and energy intake.** All statistical analysis were performed using SPSS (version 21.0) and R software (version 4.4.3). A two‐tailed *p*‐value < 0.05 was considered statistically significant.

## 3 Results

### 3.1 Participant Characteristics

Of the 4000 participants at baseline, 579 (14.5%) had pre‐existing DM (Baseline_DM) and 3421 (85.5%) did not (Baseline_Non_DM). Baseline characteristics of these two groups were shown in Table 1. The Baseline_DM group was significantly older (median 72 years, IQR 69–77) than the Baseline_Non_DM group (median 72 years, IQR 68–76) (*p* = 0.025). The two groups exhibited balanced sex distributions (49.9% versus 50.6% male). Participants with Baseline_DM had a significantly higher Charlson Comorbidity Index, higher anthropometric measures (weight, BMI, WC and waist/height), lower energy and carbohydrate intake and significantly elevated BCAA levels (all *p* < 0.05).

For body composition, the Baseline_DM group demonstrated higher ASM/ht^2^ (median 6.74 vs. 6.56 kg/m^2^, *p* < 0.001) and FMI (*p* < 0.05). Physical performance was poorer in the Baseline_DM group, with slower gait speed and longer chair stand time (both *p* < 0.01), though grip strength did not differ. In a supplementary analysis, participants lost to 14‐year follow‐up were older, had more comorbidities and exhibited poorer baseline physical performance than those retained (all *p* < 0.001; Table S5). Although no significant differences were found in key fat mass or relative muscle mass measures (e.g., PBF, ASM/weight), the retained group had higher absolute muscle mass indices (ASM/ht^2^ and lower limb‐ASM/ht^2^; both *p* < 0.05).

### 3.2 Association of Baseline Body Composition and Physical Performance With Incident DM

Cox proportional hazards models were used to assess associations between baseline measures and incident DM among 3421 participants free of DM at baseline. As shown in Table 2, in the fully adjusted model (adjusted for age, sex, PASE score, energy intake, and sociodemographic, lifestyle and clinical factors), higher adiposity measures (PBF, FMI, BMI, WC, waist‐to‐height ratio) and height‐adjusted ASM (ASM/ht^2^) were consistently associated with increased DM risk per SD increase (all HRs ≥ 1.561, all *p* < 0.001). Conversely, higher relative muscle mass (ASM/weight, ASM/BMI) was associated with a lower DM risk (HR 0.603, 95% CI 0.496–0.732 and HR 0.631, 95% CI 0.502–0.794, respectively; all *p* < 0.001). Faster gait speed also showed an inverse relationship with DM risk (HR 0.748, *p* < 0.001), while grip strength showed no significant association. Sex‐stratified analysis confirmed these patterns in both men and women. All adiposity measures and ASM/ht^2^ remained associated with increased risk, while ASM/weight and ASM/BMI were inversely related to DM risk (all *p* < 0.05).

**TABLE 2:** Association of baseline body composition and physical performance with incident DM using Cox proportional hazards regression models.^a^

	Overall (*N* = 3421)		Male (*N* = 1707)		Female (*N* = 1714)	
	HR (95% CI)	*p*	HR (95% CI)	*p*	HR (95% CI)	*p*
Adjusted model						
PBF, %	1.613 (1.326–1.961)	< 0.001	1.537 (1.256–1.880)	< 0.001	1.285 (1.064–1.553)	0.009
FMI, kg/m^2^	1.684 (1.458–1.945)	< 0.001	1.634 (1.369–1.950)	< 0.001	1.472 (1.245–1.739)	< 0.001
ASM/weight, %	0.603 (0.496–0.732)	< 0.001	0.672 (0.557–0.811)	< 0.001	0.762 (0.635–0.916)	0.004
ASM/BMI	0.631 (0.502–0.794)	< 0.001	0.722 (0.597–0.873)	< 0.001	0.826 (0.695–0.981)	0.029
ASM/ht^2^, kg/m^2^	1.561 (1.351–1.803)	< 0.001	1.448 (1.212–1.731)	< 0.001	1.400 (1.201–1.633)	< 0.001
BMI, kg/m^2^	1.659 (1.478–1.862)	< 0.001	1.729 (1.450–2.062)	< 0.001	1.581 (1.352–1.847)	< 0.001
Waist, cm	1.587 (1.403–1.794)	< 0.001	1.540 (1.283–1.848)	< 0.001	1.609 (1.355–1.911)	< 0.001
Waist/ht	1.616 (1.422–1.837)	< 0.001	1.592 (1.322–1.917)	< 0.001	1.588 (1.336–1.888)	< 0.001
Grip strength, kg	1.087 (0.908–1.302)	0.363	1.051 (0.871–1.268)	0.603	1.099 (0.930–1.298)	0.268
Gait speed, m/s	0.748 (0.654–0.857)	< 0.001	0.761 (0.626–0.925)	0.006	0.755 (0.631–0.903)	0.002
Five‐time chair stand, s	1.089 (0.971–1.222)	0.146	1.057 (0.896–1.246)	0.514	1.079 (0.913–1.274)	0.372

^a^ Adjusted model: Adjusted for age, gender, PASE score, daily caloric intake, education, living alone, smoking status, drinking status, Charlson comorbidity index, statin use and corticosteroid use. Each index was standardized using *Z*‐score.

Abbreviations: ASM, appendicular skeletal muscle mass; ASM/ht^2^, ASM/height^2^; ASM/weight, ASM/body weight × 100; BMI, body mass index; FMI, fat mass index; ht, height; PASE, Physical Activity Scale for the Elderly; PBF, percentage of body fat.

### 3.3 Sensitivity Analysis Accounting for Competing Risk of Death

Fine–Gray competing risk regression models demonstrated results consistent with the primary Cox analysis (Table 3). In the fully adjusted Model, higher baseline adiposity (e.g., PBF: subdistribution hazard ratio [sHR] 1.624, 95% CI 1.355–1.946), higher ASM/ht^2^ (sHR 1.569, 95% CI 1.364–1.805) remained significantly associated with incident DM risk (all *p* < 0.001). Conversely, higher relative muscle mass (ASM/weight: sHR 0.622, 95% CI 0.520–0.745; ASM/BMI: sHR 0.624, 95% CI 0.505–0.771) were significantly associated with a lower risk of incident DM (*p* < 0.001). For physical performance, faster gait speed (sHR 0.846, 95% CI 0.752–0.952) was also linked to a reduced risk of incident DM (*p* = 0.006).

**TABLE 3:** Association of baseline body composition and physical performance with incident DM using Fine–Gray competing risk regression models.^a^

	Overall (*N* = 3421)		Male (*N* = 1707)		Female (*N* = 1714)	
	sHR (95% CI)	*p*	sHR (95% CI)	*p*	sHR (95% CI)	*p*
Adjusted model						
PBF, %	1.624 (1.355–1.946)	< 0.001	1.528 (1.276–1.829)	< 0.001	1.306 (1.088–1.567)	0.004
FMI, kg/m^2^	1.620 (1.420–1.850)	< 0.001	1.573 (1.353–1.829)	< 0.001	1.443 (1.228–1.695)	< 0.001
ASM/weight, %	0.622 (0.520–0.745)	< 0.001	0.686 (0.576–0.816)	< 0.001	0.763 (0.642–0.906)	0.002
ASM/BMI	0.624 (0.505–0.771)	< 0.001	0.722 (0.603–0.865)	< 0.001	0.810 (0.692–0.947)	0.008
ASM/ht^2^, kg/m^2^	1.569 (1.364–1.805)	< 0.001	1.455 (1.231–1.719)	< 0.001	1.406 (1.209–1.635)	< 0.001
BMI, kg/m^2^	1.600 (1.438–1.780)	< 0.001	1.630 (1.399–1.899)	< 0.001	1.567 (1.348–1.823)	< 0.001
Waist, cm	1.516 (1.352–1.700)	< 0.001	1.458 (1.249–1.702)	< 0.001	1.571 (1.322–1.867)	< 0.001
Waist/ht	1.542 (1.373–1.731)	< 0.001	1.484 (1.270–1.734)	< 0.001	1.562 (1.321–1.846)	< 0.001
Grip strength, kg	1.156 (0.958–1.400)	0.130	1.118 (0.914–1.367)	0.279	1.097 (0.940–1.282)	0.241
Gait speed, m/s	0.846 (0.752–0.952)	0.006	0.888 (0.755–1.044)	0.151	0.813 (0.691–0.955)	0.012
Five‐time chair stand, s	1.047 (0.938–1.168)	0.412	1.027 (0.881–1.198)	0.733	1.050 (0.897–1.228)	0.546

^a^ Results were presented as subdistribution hazard ratios (sHR) with 95% confidence intervals from Fine–Gray proportional subdistribution hazards models, treating death before the onset of diabetes mellitus as a competing event. The adjusted model was controlled for age, gender, PASE score, daily caloric intake, education, living alone, smoking status, drinking status, Charlson comorbidity index, statin use and corticosteroid use. Each index was standardized using *Z*‐score.

Abbreviations: ASM, appendicular skeletal muscle mass; BMI, body mass index; ht, height; PASE, Physical Activity Scale for the Elderly; PBF, percentage of body fat; FMI, fat mass index; sHR, subdistribution hazard ratio.

### 3.4 Subgroup Analysis Among Participants With Complete 14‐Year Follow‐Up

A consistent association was also observed in the multivariable logistic regression analysis of the subgroup that completed the entire 14‐year follow‐up (*n* = 937, 130 incident DM cases; median age 69 [IQR 67–72] years, 52.1% women). As shown in the adjusted model (Table S1), findings were largely consistent with the primary Cox analysis in both direction and magnitude. Higher adiposity (PBF, FMI, BMI, WC, waist‐to‐height ratio) and ASM/ht^2^ were associated with increased DM risk (all ORs ≥ 1.639, *p* < 0.001), whereas higher relative muscle mass (ASM/weight: OR 0.633, *p* = 0.005) and faster gait speed (OR 0.788, *p* = 0.027) were associated with decreased risk. Sex‐stratified analysis showed largely comparable patterns to the overall analysis, with associations for adiposity and ASM/ht^2^ remaining significant in both sexes (all *p* < 0.05).

### 3.5 Associations of 14‐Year Changes in Body Composition and Physical Performance With Incident DM

**FIGURE 1:**
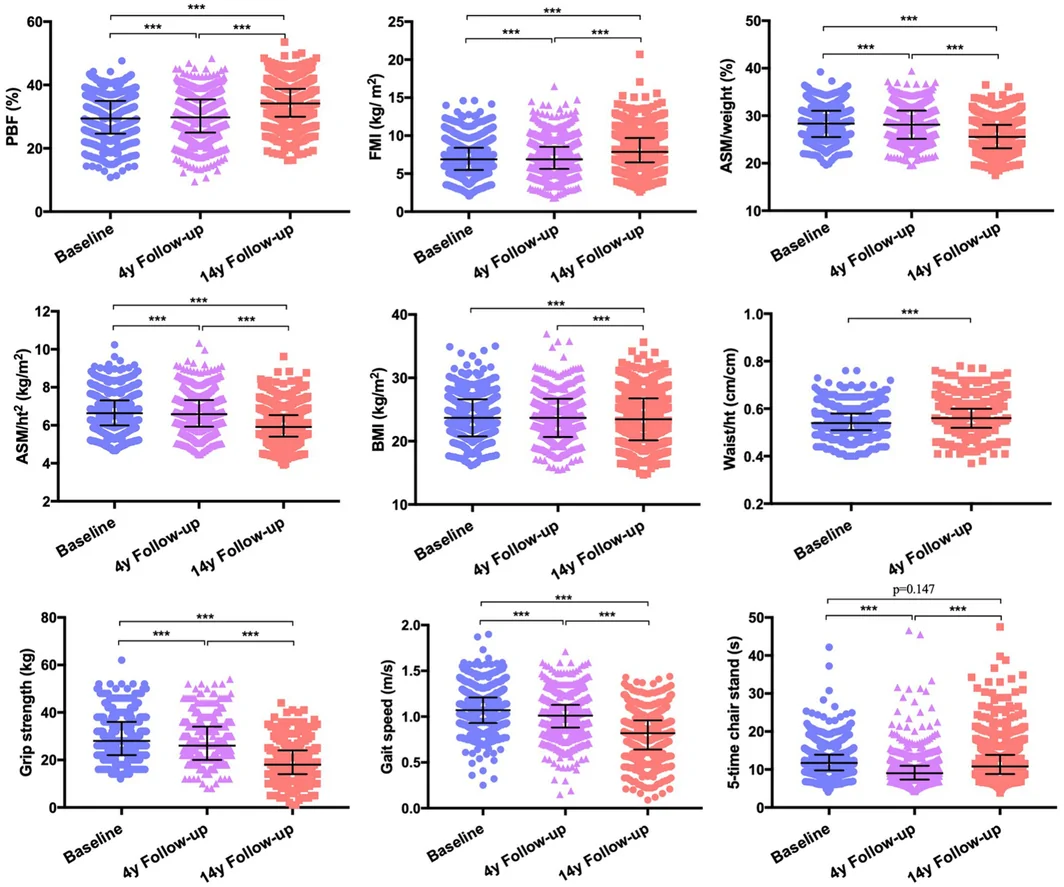
Longitudinal changes in body composition and physical performance parameters among all participants at baseline, 4‐year follow‐up and 14‐year follow‐up. Data were presented as median (interquartile range). Paired comparisons were analysed using the Wilcoxon matched‐pairs signed‐rank test. Abbreviations: FMI, fat mass index; PBF, percentage of body fat; FMI, fat mass index; ASM, appendicular skeletal muscle mass; ht, height; BMI, body mass index.

Figure 1 showed the significant changes in body composition parameters observed during the 14‐year follow‐up among 937 participants. At the 14‐year follow‐up, participants showed marked increases in adiposity indicators, including PBF (mean increase +4.30%) and FMI (+0.97 kg/m^2^) (all *p* < 0.001), while demonstrating significant reductions in muscle mass measures, particularly weight‐adjusted ASM (ASM/weight; −2.50%) and height‐adjusted ASM (ASM/ht^2^; −0.67 kg/m^2^) (−0.47 kg/m^2^) (all *p* < 0.001). Physical performance measures similarly demonstrated significant declines in both grip strength and gait speed (both *p* < 0.001), while no significant change was observed in 5‐time chair stand test.

In the fully adjusted model, which accounted for corresponding baseline values and other covariates (Table 4), attenuated loss of muscle mass (∆ASM/weight: OR 0.657, 95% CI 0.491–0.879; *p* = 0.005; ∆ASM/ht^2^: OR 0.798, 95% CI 0.644–0.990; *p* = 0.040) and increased gait speed (OR 0.792, 95% CI 0.634–0.990, *p* = 0.040) were associated with lower 14‐year DM incidence, whereas an increase in PBF was related to elevated DM risk (OR 1.509, 95% CI 1.071–2.126; *p* = 0.019). ∆ASM/BMI also showed a trend toward an inverse association with DM risk (OR: 0.795, *p* = 0.099). No significant associations were observed for ∆BMI, ∆FMI, ∆grip strength or ∆5‐chair stand time. In sex‐stratified analysis, greater preservation of ASM/weight was associated with lower DM risk in women (OR 0.650, 95% CI 0.440–0.959, *p* = 0.030), while longer chair stand time correlated with higher risk in men (OR 1.364, 95% CI 1.046–1.779, *p* = 0.022).

**TABLE 4:** Longitudinal associations of 14‐year changes in body composition and physical performance with incident DM among participants without baseline DM.^a^

	Overall (*N* = 937)		Male (*N* = 449)		Female (*N* = 488)	
	OR (95% CI)	*p*	OR (95% CI)	*p*	OR (95% CI)	*p*
Adjusted model						
∆PBF, %	1.509 (1.071–2.126)	0.019	1.454 (0.888–2.381)	0.137	1.570 (0.977–2.524)	0.062
∆FMI, kg/m^2^	1.024 (0.840–1.248)	0.815	1.113 (0.816–1.518)	0.498	0.962 (0.735–1.259)	0.779
∆ASM/weight, %	0.657 (0.491–0.879)	0.005	0.685 (0.451–1.041)	0.076	0.650 (0.440–0.959)	0.030
∆ASM/BMI	0.795 (0.605–1.044)	0.099	0.836 (0.569–1.230)	0.364	0.784 (0.554–1.109)	0.169
∆ASM/ht^2^, kg/m^2^	0.798 (0.644–0.990)	0.040	0.827 (0.597–1.146)	0.253	0.809 (0.618–1.058)	0.121
∆BMI, kg/m^2^	0.926 (0.771–1.113)	0.413	0.948 (0.713–1.262)	0.716	0.897 (0.697–1.156)	0.402
∆Grip strength, kg	0.999 (0.778–1.282)	0.992	1.080 (0.764–1.526)	0.664	0.829 (0.599–1.149)	0.261
∆Gait speed, m/s	0.792 (0.634–0.990)	0.040	0.732 (0.527–1.017)	0.063	0.887 (0.655–1.201)	0.438
∆Five‐time chair stand, s	1.103 (0.901–1.350)	0.343	1.364 (1.046–1.779)	0.022	0.877 (0.627–1.227)	0.444

^a^ Δ values were calculated as follow‐up (14‐year) measurements minus baseline measurements for all body composition and physical performance parameters. The adjusted model was controlled for age, gender, PASE score, daily caloric intake, education, smoking status, drinking status, Charlson comorbidity index, statin use and corticosteroid use, with ∆PBF and ∆ASM models additionally being adjusted for weight change. All models were further adjusted for corresponding baseline body composition and physical function measures. Each index was standardized using the *Z*‐score.

Abbreviations: ASM, appendicular skeletal muscle mass; BMI, body mass index; FMI, fat mass index; ht, height; PASE, Physical Activity Scale for the Elderly; PBF, percentage of body fat.

### 3.6 Associations of Early (0–4 Year) Prediabetes Changes in Body Composition With Long‐Term DM Risk

In an analysis restricted to 864 participants who remained diabetes‐free at both baseline and year 4, early changes (0–4 years) in body composition and physical performance were associated with long‐term DM risk (years 4–14) (Table S2). After adjustment for covariates and baseline measures, an increase in WC over the initial 4 years was associated with a higher subsequent DM risk (OR 1.428, 95% CI 1.114–1.831, *p* = 0.005). Conversely, an increase in relative muscle mass, as indicated by ∆ASM/weight (OR 0.745, 95% CI 0.590–0.940, *p* = 0.013) and ∆ASM/BMI (OR 0.792, 95% CI 0.630–0.995, *p* = 0.045), was associated with a lower DM risk. Sex‐stratified analysis revealed that increased WC was associated with a higher risk of DM in men (OR 1.859, 95% CI 1.322–2.615, *p* < 0.001), while increased ASM/weight was inversely associated with DM risk (OR 0.679, 95% CI 0.484–0.953, *p* = 0.025).

### 3.7 Mediating Roles of BCAAs on the Association Between Baseline Body Composition and 14‐Year DM Risk

**FIGURE 2:**
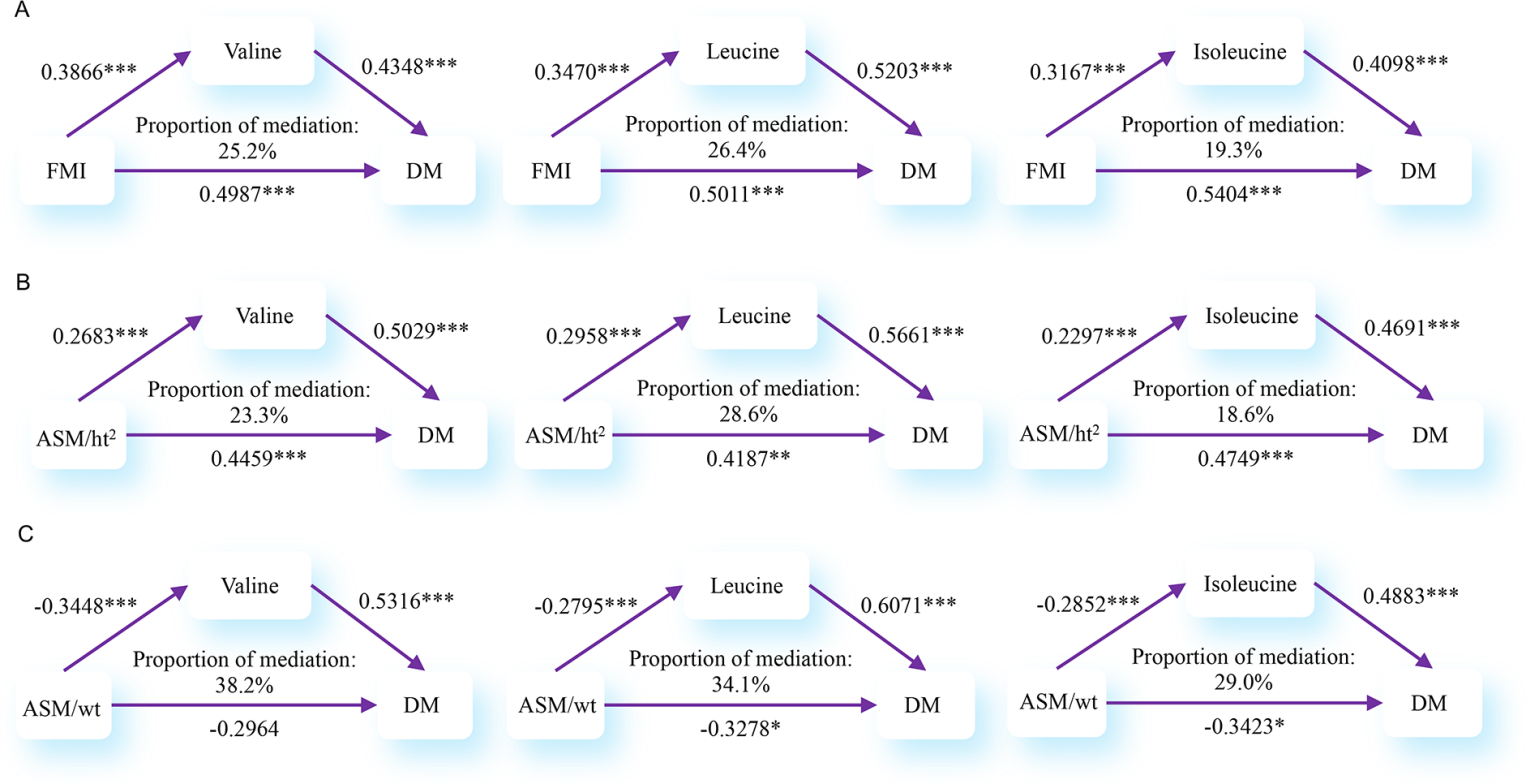
Mediation effects of serum BCAAs (valine, leucine, isoleucine) in the association between body composition and incident DM over 14 years. (A) FMI and DM, (B) ASM/ht^2^ and DM, (C) ASM/wt and DM. Standard regression coefficients were reported. All models were adjusted for age, sex, PASE score and energy intake. Abbreviations: BCAA, branched‐chain amino acid; DM, diabetes mellitus; FMI, fat mass index; ASM, appendicular skeletal muscle mass; ASM/ht^2^, ASM/height^2^; ASM/wt, ASM/weight; PASE, Physical Activity Scale for the elderly.

Mediation analysis demonstrated significant mediating effects of BCAAs in the associations between body composition and incident DM (Table S3, Figure 2). For FMI, the positive association with DM onset was partially mediated through elevated levels of valine (ACME = 0.0300, *p* < 0.001; proportion mediated = 25.2%), leucine (ACME = 0.0320, *p* < 0.001; 26.4%) and isoleucine (ACME = 0.0232, *p* < 0.001; 19.3%). For ASM/ht^2^, the mediation effects were observed for valine (ACME = 0.0146, *p* < 0.001; proportion mediated = 23.3%), leucine (ACME = 0.0179, *p* < 0.001; 28.6%) and isoleucine (ACME = 0.0116, *p* < 0.001; 18.6%). Notably, the negative association between ASM/weight and DM risk was partially mediated through decreased levels of valine (ACME = −0.0238, *p* < 0.001; 38.2%), leucine (ACME = −0.0219, *p* < 0.001; 34.1%) and isoleucine (ACME = −0.0181, *p* < 0.001; 29.0%).

Supplementary analysis confirmed that BCAA levels were consistently higher in individuals with DM compared to those without, at both baseline and the 14‐year follow‐up (all *p* < 0.001; Table S6). Higher baseline BCAA levels (per SD increase) were also associated with a high‐BCAA status 14 years later, with adjusted odds ratios ranging from 2.061 for leucine to 2.675 for isoleucine (all *p* < 0.001; Table S7).

### 3.8 Associations Between Serum BCAA Levels and Dietary Protein

**FIGURE 3:**
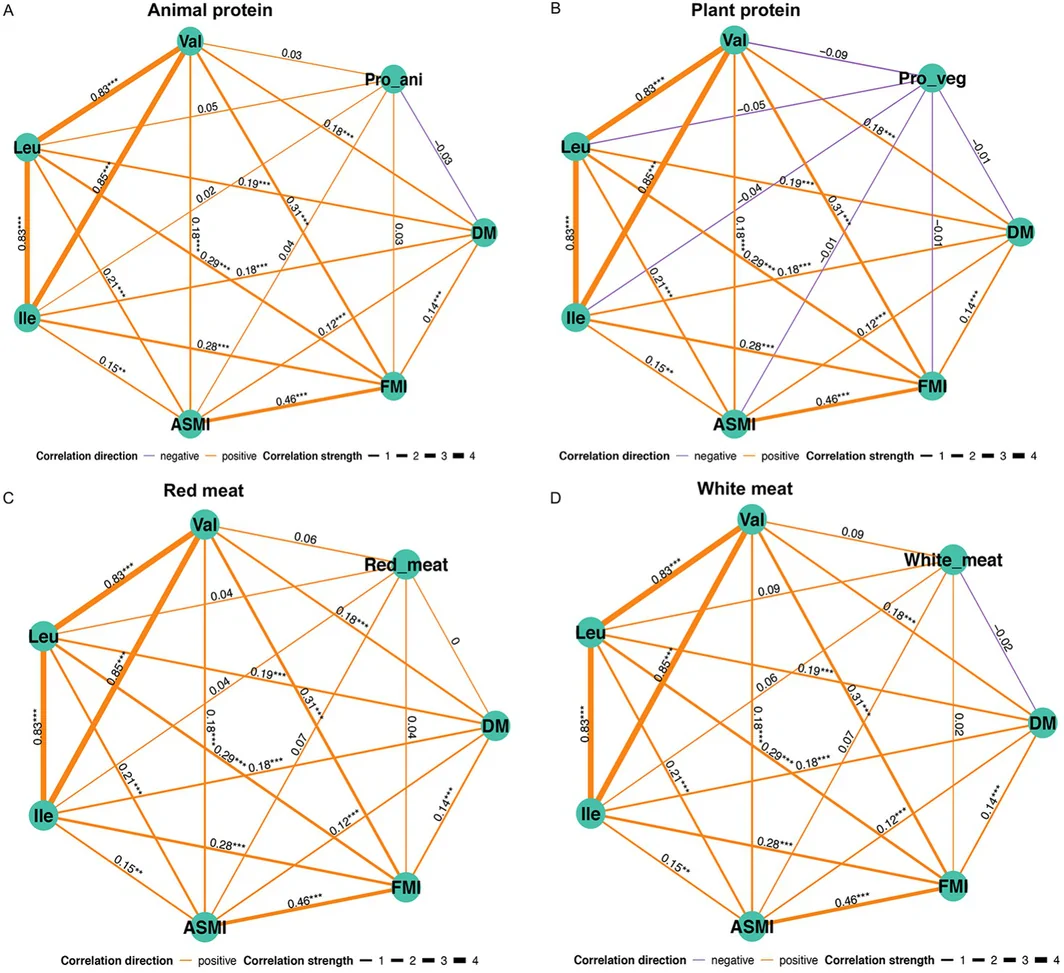
Network of partial Spearman correlations between dietary components, serum BCAAs and 14‐year incident DM. These partial Spearman correlations were adjusted for age, sex, PASE score and energy intake. Orange lines indicated positive correlations, and purple lines indicated negative correlations. The partial Spearman correlation coefficient (*ρ*) for each pair of variables was labelled adjacent to its corresponding line. The thickness of each line represented the relative visual strength of the association, which is proportional to the absolute value of *ρ*; the scale (1–4) in the legend provided a reference for this relative thickness. Statistically significant correlations after false discovery rate (FDR) correction were indicated (*p_FDR < 0.05, **p_FDR < 0.01, ***p_FDR < 0.001). Abbreviations: BCAA, branched‐chain amino acid; DM, diabetes mellitus; PASE, Physical Activity Scale for the Elderly; Pro_ani, animal protein; Pro_veg, plant protein; FMI, fat mass index; PBF, percentage of body fat; ASMI, appendicular skeletal muscle mass index (ASM/height^2^).

Given the significant mediating role of BCAAs in incident DM, we further examined relationships between serum BCAA levels and protein intake patterns (animal vs. plant sources), meat subtypes (red vs. white meat) at baseline. As shown in Table S4 and Figure 3, Spearman partial correlation analysis showed significant positive associations of animal protein with serum BCAAs (all *p* < 0.01) and of red meat with leucine and isoleucine (both *p* < 0.05). However, after adjustment for age, sex, PASE score and energy intake, all these associations were non‐significant (all *p* > 0.05). Conversely, plant protein intake showed a trend of inverse association with BCAA concentrations.

## 4 Discussion

Our study provides novel insights into the longitudinal associations between body composition and incident DM in older adults over a 14‐year follow‐up. First, we observed that higher baseline fat measures, central adiposity and height‐adjusted ASM were associated with an increased DM risk, while higher relative muscle mass (ASM/weight and ASM/BMI) and faster gait speed were linked to a lower risk. Sensitivity analysis yielded consistent results, underscoring the robustness of these associations. Second, long‐term (14‐year) muscle mass decline and fat gain elevated DM risk; early adverse changes (increased WC, decreased relative muscle mass over 0–4 years) predicted subsequent DM. Finally, serum BCAAs mediated 18.6%–38.2% of the associations between body composition and DM risk.

The divergent associations observed for different muscle mass indices warrant discussion. The positive association between ASM/ht^2^ and incident DM may reflect DXA's inability to differentiate functional muscle from intra‐ and intermuscular adipose tissue [24]. In older adults, a higher ASM/ht^2^ may thus indicate greater muscular fat infiltration, a known contributor to IR, rather than greater protective muscle mass [25, 26]. This is supported by studies linking higher ASM/ht^2^ to adverse metabolic profiles and poorer physical function [27, 28, 29]. In contrast, relative muscle mass indices were consistently associated with a lower DM risk in our cohort. This protective association is supported by evidence that ASM/weight correlates with better physical function [27, 30] and more effectively identifies sarcopenia in overweight individuals [31], likely because it accounts for metabolic load and detects “relative sarcopenia” that height‐adjusted indices may miss. Given the strong correlation of ASM/ht^2^ with BMI [30, 32], ASM/BMI also gained prominence for capturing relative sarcopenia in obesity, as reflected in major diagnostic criteria [33, 34]. Consequently, relative muscle mass indices may be more appropriate than height‐adjusted indices for assessing sarcopenic obesity and its associated DM risk in aging populations.

In addition to muscle mass, the distribution of adiposity was also a key determinant of DM risk. Consistent with the associations observed for other adiposity indices (PBF, FMI), central adiposity, as indicated by WC, was strongly and independently linked to incident DM in our cohort. The association for absolute WC was comparable to that of the waist‐to‐height ratio, underscoring the importance of central fat accumulation per se as a risk indicator [35, 36]. This aligns with clinical practice of WC, a simple and low‐cost measure, for identifying individuals with metabolic syndrome [36]. Together with the consistent associations for low relative muscle mass, these results suggest that the co‐occurrence of high central adiposity and low relative muscle mass (sarcopenic obesity) may help screen a subgroup of older adults at elevated DM risk.

Our study extends previous research by examining long‐term body composition changes. Over 14 years, declines in both ASM/weight and ASM/ht^2^ were associated with increased DM risk, while increased PBF also correlated with incident DM. Age‐related fat accumulation and redistribution, particularly from central to the lower limbs [37], suggest that declining ASM/ht^2^ likely reflects loss of functional muscle rather than decreased muscular fat infiltration [38]. These findings underscore that sarcopenic obesity (combined muscle loss and fat gain) is associated with an elevated DM risk and warrants attention in older adults. Given that 14‐year changes may include post‐diagnosis alterations, we conducted a supplementary analysis of pre‐DM changes (0–4 years) in participants free of DM at both time points. This analysis demonstrated that early increases in WC and declines in ASM/weight were already associated with future DM risk, which helps to mitigate concerns regarding reverse causality. The consistent adverse associations observed across both the long‐term and early‐change analyses strengthen the evidence for a link between shifts toward higher adiposity and lower relative muscle mass and DM risk in older adults, beginning before clinical diagnosis. These findings underscore the potential relevance of these early changes for understanding DM risk trajectories.

In addition to body composition measures, we observed that slower gait speed was associated with an increased risk of DM. While participants lost to follow‐up had poorer baseline physical performance (Table S5), potentially introducing follow‐up bias, the specific gait speed‐DM association remained significant. This association may be related to the role of lower‐body muscle function in systemic metabolism. Further studies are needed to validate the observed links between physical performance and DM risk. The primary focus of this study remains on the associations and mediating pathways involving body composition and DM.

A key finding of this study is the mediating role of baseline BCAAs in the association between body composition and DM incidence, accounting for 18.6%–38.2% of the effect. Our longitudinal BCAA data were consistent with this mediating role, showing that elevated BCAA levels were associated with DM at both baseline and the 14‐year follow‐up and that higher baseline levels correlated with a high‐BCAA state 14 years later. Prior studies have consistently shown that serum BCAAs are elevated and correlated with IR in patients with DM [14, 16], even though older adults generally have lower plasma BCAA levels [39]. These observed associations may be explained by the mechanistic pathways proposed in the literature. Previous studies suggest that IR can impair BCAA catabolism, leading to their accumulation, which in turn may exacerbate IR through various molecular pathways, potentially creating a sustained dysmetabolic state [14, 15, 16, 38, 40]. While our study did not directly assess these mechanisms or measure intramuscular fat, existing literature indicates that intramuscular fat deposition is correlated with both IR and elevated BCAA levels [11, 12, 13], suggesting a potential underlying mechanism that warrants further investigation. Importantly, in our cohort, the association between dietary animal protein intake and BCAA levels became non‐significant after multivariable adjustment, suggesting that inter‐individual BCAA differences in older adults may be more closely related to host‐specific factors than to dietary protein intake. Collectively, our mediation and longitudinal data support BCAA metabolism as a significant pathway linking body composition to DM risk in older adults, a relationship that aligns with, but does not prove, existing mechanistic models.

There exist several limitations in this study. First, DXA cannot differentiate muscle from muscular fat infiltration, potentially overestimating functional muscle mass. Second, the 14‐year change analysis may include post‐diabetes changes; however, our supplementary analysis of prediabetes (0–4 years) change analysis helps mitigate this concern. Third, the absence of repeated BCAA measurements from the same assay platform limited our ability to assess metabolic dynamics over time. Fourth, participants lost to the 14‐year follow‐up had poorer baseline physical performance, introducing potential follow‐up bias. Although primary Cox models appropriately handle censoring, residual selection bias cannot be fully ruled out; however, consistency between primary and completer analysis supports the robustness of our findings. Finally, the study focused on older Chinese adults in Hong Kong, which may limit generalizability to other populations.

## 5 Conclusions

In conclusion, this 14‐year prospective study of older adults demonstrated that both baseline levels and adverse longitudinal changes in body composition, specifically higher adiposity and lower relative muscle mass (ASM/weight and ASM/BMI), were associated with an increased risk of developing DM. Notably, the association between adverse body composition and DM risk was partially mediated by elevated levels of BCAAs. These findings underscore the potential importance of assessing both fat and muscle mass, particularly using relative muscle mass indices, and of considering metabolic pathways involving BCAAs in understanding DM risk in the aging population.

## Funding

This study was funded by the National Institutes of Health R01 grant [AR049439‐01A1] and the Research Grants Council Earmarked grant [CUHK4101/02 M].

## Ethics Statement

The study was conducted according to the guidelines of the Declaration of Helsinki and approved by the joint Chinese University of Hong Kong and New Territories East Cluster Clinical Research Ethics Committees in Hong Kong (Reference number [2003.102]). Written informed consent was obtained from all participants.

## Conflicts of Interest

The authors declare no conflicts of interest.

## Supporting information


**Table S1:** Long‐term association of baseline body composition and physical performance with incident DM after 14 years of follow‐up^a^.
**Table S2:** Association of pre‐DM changes (0–4 years) in body composition and physical performance with incident DM during long‐term follow‐up (4–14 years) among participants free of DM at baseline and year 4^a^.
**Table S3:** Mediation effects of serum BCAAs on the associations between body composition parameters and incident DM^a^.
**Table S4:** Correlations of baseline dietary protein and meat sources with serum BCAA levels^a^.
**Table S5:** Baseline characteristics of participants included in the 14‐year analysis versus those lost to follow‐up^a^.
**Table S6:** Serum BCAA levels at baseline and 14‐year follow‐up according to diabetes status.
**Table S7:** Risk of high serum BCAA levels at 14‐year follow‐up by baseline BCAA levels.

## Data Availability

The data that support the findings of this study are available from the corresponding author upon reasonable request.
