# Supplementary material for: Associations of Body Composition and Physical Function With Incident Diabetes in Older Adults: A 14‐Year Prospective Cohort Study
Source: J Cachexia Sarcopenia Muscle. 2026 Apr 20;17(2):e70297. doi: 10.1002/jcsm.70297 (PMC13093723; doi:10.1002/jcsm.70297)
Supplement: Supplementary file 1 — Table S1: Long‐term association of baseline body composition and physical performance with incident DM after 14 years of follow‐upa. Table S2: Association of pre‐DM changes (0–4 years) in body composition and physical performance with incident DM during long‐term follow‐up (4–14 years) among participants free of DM at baseline and year 4a. Table S3: Mediation effects of serum BCAAs on the associations between body composition parameters and incident DMa. Table S4: Correlations of baseline dietary protein and meat sources with serum BCAA levelsa. Table S5: Baseline characteristics of participants included in the 14‐year analysis versus those lost to follow‐upa. Table S6: Serum BCAA levels at baseline and 14‐year follow‐up according to diabetes status. Table S7: Risk of high serum BCAA levels at 14‐year follow‐up by baseline BCAA levels. [file JCSM-17-e70297-s001.docx]

**Supporting information**

**Associations of Body Composition and Physical Function with Incident Diabetes in Older Adults: A 14-Year Prospective Cohort Study**

Ting Zhang^1, 2^, Shuyi Li^2^, Yafei, Wu^2^, Jason Leung^3^, Alice Pik-Shan Kong^2, 4^, Amany K. Elshorbagy^5, 6^, T W Auyeung^7^, Timothy Kwok^2, 3^

^1^Department of Geriatrics, Ren Ji Hospital, Shanghai Jiao Tong University School of Medicine, Shanghai 200127, China

^2^Department of Medicine and Therapeutics, Faculty of Medicine, The Chinese University of Hong Kong, Hong Kong, China

^3^Jockey Club Centre for Osteoporosis Care and Control, The Chinese University of Hong Kong, Hong Kong, China

^4^Hong Kong Institute of Diabetes and Obesity, Prince of Wales Hospital, The Chinese University of Hong Kong, Shatin, Hong Kong, China

^5^Department of Pharmacology, University of Oxford, Oxford, UK

^6^Department of Physiology, Faculty of Medicine, University of Alexandria, Alexandria, Egypt

^7^Jockey Club Institute of Ageing, The Chinese University of Hong Kong, Hong Kong, China

*Corresponding author:

Timothy Kwok

MD, Professor

Department of Medicine and Therapeutics, Prince of Wales Hospital, The Chinese University of Hong Kong, Shatin, Hong Kong, China.

E-mail: tkwok@cuhk.edu.hk; Tel.: +852-2632-3128; Fax: +852-2637-3852

**Table S1** Long-term association of baseline body composition and physical performance with incident DM after 14 years of follow-up ^a^.

|  | **Overall (N=937)** |  | **Male (N=449)** |  | **Female (N=488)** |  |
| --- | --- | --- | --- | --- | --- | --- |
|  | **OR (95% CI)** | ***p*** | **OR (95% CI)** | ***p*** | **OR (95% CI)** | ***p*** |
| Adjusted Model |  |  |  |  |  |  |
| PBF, % | 1.885 (1.379-2.578) | <0.001 | 1.476 (1.085-2.009) | 0.013 | 1.560 (1.168-2.085) | 0.003 |
| FMI, kg/ m^2^ | 1.855 (1.464-2.350) | <0.001 | 1.555 (1.169-2.068) | 0.002 | 1.750 (1.333-2.298) | <0.001 |
| ASM/weight, % | 0.633 (0.460-0.872) | 0.005 | 0.717 (0.529-0.970) | 0.031 | 0.788 (0.598-1.037) | 0.089 |
| ASM/BMI | 0.791 (0.546-1.147) | 0.217 | 0.887 (0.660-1.192) | 0.425 | 0.866 (0.657-1.141) | 0.307 |
| ASM/ht^2^, kg/m^2^ | 1.735 (1.355-2.221) | <0.001 | 1.412 (1.071-1.863) | 0.014 | 1.618 (1.243-2.106) | <0.001 |
| BMI, kg/m^2^ | 1.715 (1.417-2.076) | <0.001 | 1.612 (1.217-2.136) | <0.001 | 1.804 (1.378-2.361) | <0.001 |
| Waist, cm | 1.697 (1.390-2.072) | <0.001 | 1.634 (1.218-2.192) | 0.001 | 1.785 (1.344-2.371) | <0.001 |
| Waist/ht | 1.639 (1.344-1.999) | <0.001 | 1.563 (1.166-2.095) | 0.003 | 1.731 (1.311-2.285) | <0.001 |
| Grip strength, kg | 0.963 (0.709-1.307) | 0.809 | 0.865 (0.650-1.153) | 0.323 | 1.163 (0.895-1.511) | 0.258 |
| Gait speed, m/s | 0.788 (0.638-0.973) | 0.027 | 0.865 (0.641-1.166) | 0.342 | 0.716 (0.539-0.951) | 0.021 |
| 5-times chair stand, s | 1.013 (0.839-1.224) | 0.892 | 0.947 (0.712-1.260) | 0.710 | 1.023 (0.781-1.340) | 0.868 |

^a^ Adjusted Model: Adjusted for age, gender, PASE score, daily caloric intake, education, living alone, smoking status, drinking status, Charlson comorbidity index, statin use and corticosteroid use. All indexes were standardized using Z-score. Abbreviations: PASE, physical activity scale for the elderly; PBF, percent of body fat; FMI, fat mass index; BMI, body mass index; ht, height; ASM, appendicular skeletal muscle mass.

**Table S2** Association of pre-DM changes (0-4 years) in body composition and physical performance with incident DM during long-term follow-up (4-14 years) among participants free of DM at baseline and year 4 ^a^.

|  | **Overall (N=864)** |  | **Male (N=417)** |  | **Female (N=447)** |  |
| --- | --- | --- | --- | --- | --- | --- |
|  | **OR (95% CI)** | ***p*** | **OR (95% CI)** | ***p*** | **OR (95% CI)** | ***p*** |
| Adjusted Model |  |  |  |  |  |  |
| ∆PBF, % | 1.174 (0.928-1.486) | 0.182 | 1.273 (0.909-1.783) | 0.161 | 1.064 (0.760-1.490) | 0.717 |
| ∆FMI, kg/ m^2^ | 1.178 (0.945-1.469) | 0.146 | 1.297 (0.936-1.797) | 0.119 | 1.074 (0.788-1.465) | 0.651 |
| ∆ASM/weight, % | 0.745 (0.590-0.940) | 0.013 | 0.679 (0.484-0.953) | 0.025 | 0.818 (0.593-1.128) | 0.220 |
| ∆ASM/BMI | 0.792 (0.630-0.995) | 0.045 | 0.734 (0.524-1.027) | 0.071 | 0.860 (0.630-1.175) | 0.344 |
| ∆ASM/ht^2^, kg/m^2^ | 1.038 (0.836-1.289) | 0.736 | 0.963 (0.701-1.323) | 0.816 | 1.093 (0.810-1.475) | 0.560 |
| ∆BMI, kg/m^2^ | 1.234 (0.994-1.533) | 0.056 | 1.337 (0.970-1.844) | 0.076 | 1.145 (0.846-1.551) | 0.381 |
| ∆Waist, cm | 1.428 (1.114-1.831) | 0.005 | 1.859 (1.322-2.615) | <0.001 | 1.141 (0.818-1.591) | 0.438 |
| ∆Waist/ht | 1.374 (1.076-1.755) | 0.011 | 1.825 (1.305-2.552) | <0.001 | 1.103 (0.795-1.531) | 0.557 |
| ∆Grip strength, kg | 1.024 (0.802-1.307) | 0.849 | 1.037 (0.731-1.471) | 0.839 | 1.023 (0.722-1.449) | 0.899 |
| ∆Gait speed, m/s | 0.910 (0.708-1.170) | 0.463 | 0.898 (0.619-1.302) | 0.570 | 0.930 (0.661-1.308) | 0.675 |
| ∆5-times chair stand, s | 1.059 (0.819-1.370) | 0.662 | 1.289 (0.828-2.005) | 0.261 | 0.972 (0.672-1.406) | 0.879 |

^a^ The analysis included participants who were free of diabetes mellitus (DM) at both baseline and the 4-year follow-up, and who underwent body composition/physical performance assessments at both time points. Incident DM was assessed from year 4 to year 14, ensuring that all analyzed changes occurred before any DM diagnosis in the study period. Δ values were calculated as follow-up (4-year) measurements minus baseline measurements for all body composition and physical performance parameters. All models were adjusted for age, gender, PASE score and daily caloric intake; and were further adjusted for their corresponding baseline body composition and physical function measures. All variables were standardized as Z-scores. Abbreviations: PASE, physical activity scale for the elderly; PBF, percent of body fat; BMI, body mass index; ht, height; FMI, fat mass index; ASM, appendicular skeletal muscle mass.

**Table S3** Mediation effects of serum BCAAs on the associations between body composition parameters and incident DM ^a^.

|  | **Valine** |  | **Leucine** |  | **Iso-Leucine** |  |
| --- | --- | --- | --- | --- | --- | --- |
|  | **Estimate (95% CI)** | **P-value** | **Estimate (95% CI)** | **P-value** | **Estimate (95% CI)** | **P-value** |
| FMI |  |  |  |  |  |  |
| ACME (average) | 0.0300 (0.0151, 0.0464) | <0.001 | 0.0320 (0.0179, 0.0482) | <0.001 | 0.0232 (0.0117, 0.0365) | <0.001 |
| ADE (average) | 0.0891 (0.0362, 0.1533) | <0.001 | 0.0890 (0.0360, 0.1523) | <0.001 | 0.0967 (0.0437, 0.1608) | <0.001 |
| Total Effect | 0.1191 (0.0635, 0.1846) | <0.001 | 0.1210 (0.0648, 0.1868) | <0.001 | 0.1198 (0.0643, 0.1853) | <0.001 |
| Prop. Mediated | 25.2% (12.1%, 47.4%) | <0.001 | 26.4% (14.2%, 48.0%) | <0.001 | 19.3% (9.4%, 36.1%) | <0.001 |
| ASM/ht^2^ |  |  |  |  |  |  |
| ACME (average) | 0.0146 (0.0076, 0.0230) | <0.001 | 0.0179 (0.0099, 0.0274) | <0.001 | 0.0116 (0.0058, 0.0187) | <0.001 |
| ADE (average) | 0.0479 (0.0199, 0.0744) | <0.001 | 0.0446 (0.0166, 0.0702) | 0.001 | 0.0507 (0.0233, 0.0765) | <0.001 |
| Total Effect | 0.0625 (0.0350, 0.0891) | <0.001 | 0.0625 (0.0349, 0.0884) | <0.001 | 0.0623 (0.0350, 0.0884) | <0.001 |
| Prop. Mediated | 23.3% (11.9%, 47.5%) | <0.001 | 28.6% (15.6%, 55.6%) | <0.001 | 18.6% (9.1%, 37.3%) | <0.001 |
| ASM/weight |  |  |  |  |  |  |
| ACME (average) | -0.0238 (-0.0366, -0.0129) | <0.001 | -0.0219 (-0.0339, -0.0118) | <0.001 | -0.0181 (-0.0288, -0.0093) | <0.001 |
| ADE (average) | -0.0384 (-0.0894, 0.0057) | 0.095 | -0.0423 (-0.0931, 0.0028) | 0.067 | -0.0444 (-0.0946, 0.0002) | 0.049 |
| Total Effect | -0.0622 (-0.1150, -0.0161) | 0.004 | -0.0642 (-0.1175, -0.0168) | 0.004 | -0.0625 (-0.1142, -0.0164) | 0.003 |
| Prop. Mediated | 38.2% (17.6%, 129.1%) | 0.004 | 34.1% (16.4%, 111.6%) | 0.004 | 29.0% (12.9%, 96.7 %) | 0.003 |

^a^ Standard ACME, ADE, Total Effect and Prop. Mediated were reported. All models were adjusted for age, sex, PASE score and energy intake. Abbreviations: BCAA, branched-chain amino acid; DM, diabetes mellitus; ACME, average causal mediation effect; ADE, average direct effect; Total Effect, sum of ACME and ADE; PASE, physical activity scale for the elderly; FMI, fat mass index; ASM, appendicular skeletal muscle mass; ASM/ht^2^, ASM/height^2^; ASM/wt, ASM/weight.

**Table S4** Correlations of baseline dietary protein and meat sources with serum BCAA levels ^a^.

| Variables | **Valine** |  | **Leucine** |  | **Isoleucine** |  |
| --- | --- | --- | --- | --- | --- | --- |
|  | **r** | ***p*** | **r** | ***p*** | **r** | ***p*** |
| Animal protein |  |  |  |  |  |  |
| Unadjusted | 0.111 | 0.001 | 0.192 | <0.001 | 0.139 | <0.001 |
| Adjusted | 0.031 | 0.543 | 0.050 | 0.333 | 0.023 | 0.650 |
| Plant protein |  |  |  |  |  |  |
| Unadjusted | 0.039 | 0.259 | 0.082 | 0.018 | 0.056 | 0.102 |
| Adjusted | -0.090 | 0.082 | -0.054 | 0.295 | -0.038 | 0.466 |
| Red meat |  |  |  |  |  |  |
| Unadjusted | 0.052 | 0.128 | 0.069 | 0.046 | 0.070 | 0.042 |
| Adjusted | 0.060 | 0.241 | 0.044 | 0.391 | 0.036 | 0.480 |
| White meat |  |  |  |  |  |  |
| Unadjusted | 0.040 | 0.242 | 0.056 | 0.105 | 0.022 | 0.514 |
| Adjusted | 0.087 | 0.091 | 0.092 | 0.072 | 0.056 | 0.281 |

^a^ Data were presented as Spearman correlation coefficients (r) and p-values. Adjusted analysis controlled for age, sex, PASE score and energy intake using Spearman partial correlation. PASE, physical activity scale for the elderly; BCAA, branched-chain amino acid.

**Table S5** Baseline characteristics of participants included in the 14-year analysis versus those lost to follow-up ^a^.

|  | **Participants included (n=937)** | **Participants without 14y follow-up information (n=2484)** | ***P*** |
| --- | --- | --- | --- |
| Age, year | 69 (67-72) | 73 (69-77) | <0.001 |
| Men, % | 47.9 | 50.6 | 0.155 |
| Education, % |  |  | <0.001 |
| No education | 16.4 | 22.7 |  |
| Primary or below | 47.8 | 51.7 |  |
| Secondary or above | 35.8 | 25.7 |  |
| Living alone, % | 7.6 | 11.8 | 0.001 |
| Smoking status, Current smoking, % | 4.4 | 8.3 | <0.001 |
| Drinking status in the past 12 months, ≥ 28 drinks/week, % | 0.1 | 0.2 | 0.452 |
| Charlson Comorbidity Index | 4 (3-4) | 4 (4-5) | <0.001 |
| BMI, kg/m^2^ | 23.5 (21.7-25.6) | 23.4 (21.3-25.7) | 0.170 |
| Waist circumference, cm | 85.1 (80.0-91.4) | 86.4 (80.0-92.7) | 0.010 |
| PASE score | 93.7 (69.1-119.7) | 84.7 (59.7-110.4) | <0.001 |
| Energy intake, kcal/d | 1762.5 (1424.2-2247.3) | 1772.5 (1417.6-2217.7) | 0.654 |
| Carbohydrate intake, g/d | 250.5 (201.1-310.9) | 247.2 (200.6-309.5) | 0.572 |
| Fat intake, g/d | 53.0 (41.3-70.4) | 53.7 (41.3-71.0) | 0.770 |
| Total protein intake, g/d | 71.5 (55.2-96.2) | 69.7 (51.7-93.2) | 0.017 |
| Statin use, % | 5.55 | 5.07 | 0.575 |
| Corticosteroid use, % | 0.85 | 1.45 | 0.168 |
| Valine, µmol/L | 287.7 (259.2-320.9) | 284.2 (255.6-317.1) | 0.135 |
| Leucine, µmol/L | 146.1 (131.3-161.6) | 144.1 (129.0-161.7) | 0.120 |
| Isoleucine, µmol/L | 78.5 (69.1-87.7) | 77.5 (68.6-87.3) | 0.347 |
| PBF, % | 29.4 (24.6-35.0) | 29.2 (24.1-35.3) | 0.833 |
| FMI, kg/ m^2^ | 6.88 (5.49-8.40) | 6.84 (5.28-8.66) | 0.532 |
| ASM/weight, % | 28.35 (25.53-31.03) | 28.19 (25.20-30.97) | 0.268 |
| ASM/BMI | 0.692 (0.585-0.825) | 0.694 (0.575-0.819) | 0.087 |
| ASM/ht^2^, kg/m^2^ | 6.64 (5.99-7.32) | 6.53 (5.84-7.22) | 0.003 |
| Lower limb-ASM/ht^2^, kg/m^2^ | 4.96 (4.50-5.44) | 4.86 (4.37-5.37) | 0.001 |
| Grip strength, kg | 28 (22-36) | 26 (22-34) | <0.001 |
| Gait speed, m/s | 1.07 (0.93-1.21) | 1.00 (0.85-1.14) | <0.001 |
| 5-times chair stand, s | 11.72 (9.77-13.89) | 12.39 (10.40-14.98) | <0.001 |

^a^ Median (interquartile range) or Mean ± Standard Deviation for continuous variables and percentage (%) for categorical variables were shown. Abbreviations: BMI, body mass index; PASE, physical activity scale for the elderly; PBF, percent of body fat; FMI, Fat mass index; ASM, Appendicular skeletal muscle mass; ASM/ht^2^, Appendicular skeletal muscle index/height²; ASM/weight, ASM/body weight ×100.

**Table S6** Serum BCAA levels at baseline and 14-year follow-up according to diabetes status.

| **Assessment time point** | **Baseline (LC/MS)** | |  | **14-year follow-up**  **(¹H NMR spectroscopy)** | |  |
| --- | --- | --- | --- | --- | --- | --- |
|  | **Non_DM** | **DM** | **P value** | **Non_DM** | **DM** | **P value** |
| **Total BCAA** | 506.45 (457.67-562.42) | 546.69 (500.88-600.55) | <0.001 | 412.03 (368.67-455.66) | 458.24 (411.31-497.04) | <0.001 |
| **Valine** | 283.85 (257.04-316.91) | 304.64 (279.65-338.39) | <0.001 | 234.98 (212.95-260.06) | 258.01 (235.47-278.77) | <0.001 |
| **Leucine** | 144.79 (129.19-159.94) | 155.57 (141.43-171.32) | <0.001 | 122.85 (108.82-136.66) | 137.89 (121.15-150.92) | <0.001 |
| **Isoleucine** | 77.03 (68.09-86.80) | 83.02 (76.04-92.64) | <0.001 | 51.33 (43.76-60.28) | 59.98 (52.76-66.29) | <0.001 |

Abbreviations: DM, diabetes mellitus; LC/MS, liquid chromatography-mass spectrometry; ¹H NMR, proton nuclear magnetic resonance.

**Table S7 Risk of high serum BCAA levels at 14-year follow-up by baseline BCAA levels.**

|  | **Model 1** |  | **Model 2** |  | **Model 3** |  |
| --- | --- | --- | --- | --- | --- | --- |
|  | **OR (95% CI)** | **P value** | **OR (95% CI)** | **P value** | **OR (95% CI)** | **P** |
| **Outcome:** High total BCAA level at Year 14 (≥ median) | | | | | | |
| **Baseline Total BCAA** | 2.416 (2.020-2.891) | <0.001 | 2.357 (1.946-2.854) | <0.001 | 2.424 (1.994-2.947) | <0.001 |
| **Outcome:** High total valine level at Year 14 (≥ median) | | | | | | |
| **Baseline Valine** | 2.247 (1.887-2.676) | <0.001 | 2.223 (1.848-2.675) | <0.001 | 2.255 (1.870-2.719) | <0.001 |
| **Outcome:** High total leucine level at Year 14 (≥ median) | | | | | | |
| **Baseline Leucine** | 2.173 (1.829-2.582) | <0.001 | 2.028 (1.684-2.443) | <0.001 | 2.061 (1.705-2.492) | <0.001 |
| **Outcome:** High total isoleucine level at Year 14 (≥ median) | | | | | | |
| **Baseline Isoleucine** | 2.952 (2.425-3.595) | <0.001 | 2.616 (2.133-3.209) | <0.001 | 2.675 (2.170-3.297) | <0.001 |

**^a^** Model 1 raw model. Model 2 Adjusted for age, gender, PASE score and daily caloric intake. Model 3 Adjusted for Model 2 plus education, living alone, smoking status, drinking status, Charlson comorbidity index, statin use and corticosteroid use. All indexes were standardized using Z-score. Abbreviations: PASE, physical activity scale for the elderly; BCAA, Branched-chain amino acid.
